# The Eye as the Window to the Heart: Optical Coherence Tomography Angiography Biomarkers as Indicators of Cardiovascular Disease

**DOI:** 10.3390/jcm13030829

**Published:** 2024-01-31

**Authors:** Rebecca L. Kellner, Alon Harris, Lauren Ciulla, Giovanna Guidoboni, Alice Verticchio Vercellin, Francesco Oddone, Carmela Carnevale, Mohamed Zaid, Gal Antman, Jeffrey T. Kuvin, Brent Siesky

**Affiliations:** 1 Department of Ophthalmology, Icahn School of Medicine at Mount Sinai, New York, NY 10029, USA; rebecca.kellner@icahn.mssm.edu (R.L.K.); alon.harris@mssm.edu (A.H.); alice.verticchio@mssm.edu (A.V.V.); antmangal@gmail.com (G.A.); 2 Department of Ophthalmology and Visual Science, The University of Chicago, Chicago, IL 60637, USA; lmciulla@gmail.com; 3 Maine College of Engineering and Computing, University of Maine, Orono, ME 04469, USA; giovanna.guidoboni@maine.edu; 4 Glaucoma Unit, IRCCS—Fondazione Bietti, 00198 Rome, Italy; oddonef@gmail.com (F.O.); carmela.carnevale@fondazionebietti.it (C.C.); 5 Graduate School of Biomedical Science and Engineering, University of Maine, Orono, ME 04469, USA; mohamed.zaid1@maine.edu; 6 Department of Ophthalmology, Rabin Medical Center, Petah Tikva 4941492, Israel; 7 Faculty of Medicine, Tel Aviv University, Tel Aviv 69978, Israel; 8 Zucker School of Medicine at Hofstra/Northwell Health, Manhasset, NY 11549, USA; jkuvin@northwell.edu

**Keywords:** optical coherence tomography angiography, cardiovascular diseases, imaging

## Abstract

Alterations in microvasculature represent some of the earliest pathological processes across a wide variety of human diseases. In many organs, however, inaccessibility and difficulty in directly imaging tissues prevent the assessment of microvascular changes, thereby significantly limiting their translation into improved patient care. The eye provides a unique solution by allowing for the non-invasive and direct visualization and quantification of many aspects of the human microvasculature, including biomarkers for structure, function, hemodynamics, and metabolism. Optical coherence tomography angiography (OCTA) studies have specifically identified reduced capillary densities at the level of the retina in several eye diseases including glaucoma. This narrative review examines the published data related to OCTA-assessed microvasculature biomarkers and major systemic cardiovascular disease. While loss of capillaries is being established in various ocular disease, pilot data suggest that changes in the retinal microvasculature, especially within the macula, may also reflect small vessel damage occurring in other organs resulting from cardiovascular disease. Current evidence suggests retinal microvascular biomarkers as potential indicators of major systemic cardiovascular diseases, including systemic arterial hypertension, atherosclerotic disease, and congestive heart failure.

## 1. Introduction

Cardiovascular disease (CVD) is a leading cause of death globally, accounting for approximately 32% of all fatalities, with myocardial infarctions and strokes accounting for 85% [1]. CVDs are defined as a group of disorders of the heart and its vessels, and include coronary heart disease (CHD), cerebrovascular disease, peripheral arterial disease, rheumatic heart disease, congenital heart disease, deep vein thrombosis, and pulmonary embolism [1]. Despite its high prevalence and significant impact on society, developing novel accurate non-invasive diagnostic and prognostic methods for managing systemic vascular disease remains challenging due to tissue inaccessibility and the related challenges in the direct visualization of small blood vessels.

The eye’s microvasculature is singularly unique in that it can be non-invasively and directly visualized, imaged, and quantified. The integrity and function of the retinal tissue are dependent on adequate perfusion via this microcirculatory network and well-controlled blood flow [2]. Due to the accessibility of the eye’s vascular beds, the use of eye vasculature to assess pathological changes within retinal microvascular beds as an indicator for systemic vascular pathophysiology has been investigated in pilot work [2,3,4,5,6]. Specifically, changes in retinal microvasculature, particularly at the level of the macula, and variations in the thickness and layering of the retina have been associated with various aspects of cardiovascular health [2,7,8,9].

**Figure 1:**
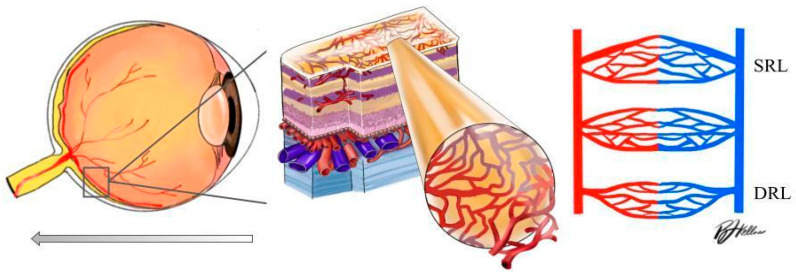
**Left**: schematic diagram of the human eye with an enlargement of the retinal tissue. **Right**: schematic diagram of the significant vascular layers of the retina discussed in this paper (DRL: deep retinal layer; SRL: superficial retinal layer).

Optical coherence tomography angiography (OCTA) is an advanced ocular imaging platform that provides structural biomarkers and a three-dimensional assessment of large and small retinal capillaries within layers of the retina and choroid. OCTA provides information related to the optic nerve region, measuring VD at the level of the radial peripapillary capillary slab (from the internal limiting membrane to the nerve fiber layer). OCTA is also able to provide a visualization of the macular superficial capillary plexus (SCP) and deep capillary plexus (DCP), which allows for a more detailed study of retinal microvascular changes at their respective layers [10] (Figure 1). OCTA can both delineate the foveal avascular zone (FAZ) and produce depth-resolved images of the superficial and deep vascular plexuses, known as the superficial and deep retinal layer (SRL and DRL) [11,12]. To date, OCTA pilot research has identified a loss of capillary density at the level of the macula, corresponding to changes in vascular density (VD) and signal intensity in hypertension and rhegmatogenous retinal detachment [13,14].

**Figure 2:**
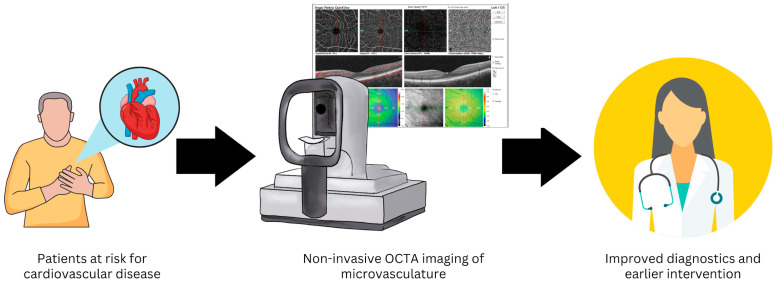
Non-invasive optical coherence tomography angiography (OCTA) imaging of the retinal microvasculature may provide a method to improve diagnostics and allow earlier intervention in patients at risk for cardiovascular disease.

The non-invasive nature and wide accessibility of OCTA in outpatient clinical settings provide high potential for assessing the ocular microvasculature as a window into the health of the systemic cardiovascular system (Figure 2). The purpose of this research article is to analyze the available data related to OCTA-assessed retinal (macular and peripapillary where available) microvascular capillaries as potential prognostic indicators for systemic cardiovascular diseases, including systemic hypertension, atherosclerotic disease, and congestive heart failure.

## 2. Methods

This analysis includes all published data related to OCTA-assessed microvascular VD biomarkers and major systemic cardiovascular disease. To achieve this, a comprehensive search was conducted through 1 December 2023 using PubMed, Embase, Ovid, Scopus, and Trip searches including the following keywords with AND/OR statements: cardiovascular disease, myocardial infarction, coronary artery disease, ischemic heart disease, optical coherence tomography, optical coherence tomography angiography, enhanced depth imaging, perfusion, blood flow, vascular density, retina, and choroidal imaging. Articles were screened for relevance, and reference lists of relevant articles were also searched and cross-referenced for other relevant articles. Data were collected and organized using Microsoft Word (version 16.30), Microsoft Excel (version 16.30), and EndNote (X8.2). 

## 3. Results and Discussion

### 3.1. Systemic Arterial Hypertension

Systemic arterial hypertension is highly prevalent and remains the predominant risk factor for cardiovascular disease. Within the eye, elevated blood pressure results in hypertensive retinopathy characterized by several retinal microvascular changes [15]. Various population-based studies have demonstrated that smaller retinal arteriolar calibers and larger retinal venular calibers precede the clinical stage of systemic hypertension and can predict up to a 5-year risk of clinical hypertension in initially normotensive individuals [5,6]. A study measuring capillary microvasculature by invasive fundus fluorescein angiography showed an increase in the perifoveal inter-capillary area and a decrease in capillary blood velocity in patients with hypertension [16]. 

Recent studies have used OCTA to investigate retinal vascularity in patients with systemic hypertension, finding a significant reduction in VD in hypertensive patients [17,18]. As seen in Table 1, multiple studies demonstrate a decrease in VD in both the deep and superficial vascular plexi [13,17,18,19,20,21,22,23,24,25,26,27]. As shown in Table 1, different studies reported the VD values of both the parafoveal region alone and the entire macular region analyzed, with VD values averaged as a mean. Lee et al. [13] examined the SCP and found that in patients with chronic hypertension and hypertensive retinopathy, there was a reduction in the superficial macular VD and perfusion density, and an increase in the FAZ, compared to those in their normotensive control group [13]. A similar study by Peng et al. [19] found that the deep vascular plexus (DVP) VD was significantly decreased in essential hypertensive patients with and without hypertensive retinopathy compared to that of controls. Of note, VD in the superficial vascular plexus (SVP) of patients with hypertensive retinopathy was significantly decreased compared to that of patients with essential hypertension without hypertensive retinopathy and that of the control group [19]. 

A larger cohort study by Chua et al. [20] examined the SVP and DVP and demonstrated that patients with poorly controlled blood pressure (BP ≥ 140/90 mmHg) had reduced DRL VD [20]. Bridging this concept, Donati et al. [21] showed a significant reduction in the VD of the DRL and an enlargement of the deep FAZ area in hypertensive patients, thus suggesting the presence of an early modification of the microvascular network at the level of the deep retinal plexus [21]. Parafoveal VD in the choriocapillaris, DCP, and total capillary plexus was also found to be decreased in hypertensive patients versus those of controls [22]. Conversely, no significant difference was found in the SCP and middle capillary plexus between hypertensive and control patients [22]. Similar results were obtained in another study in which there was a significant decrease in VD between hypertensive and control eyes in the DRL, but no significant difference in the VD of the SRL [23]. Other studies, however, found statistically significant capillary dropout in both the DRL and SRL in patients with hypertension [24,25]. Significant capillary dropout in both the SRL and DRL has also been reported in patients with recent hypertensive crises (systolic BP > 180 mmHg and diastolic BP > 110 mmHg) within 7 days [25]. These results point to a consistency of capillary dropout in poorly controlled blood pressure; however, exact mechanistic pathways and the level of tissue insult require a targeted approach for confirmation in larger controlled samples.

The length of hypertensive diagnosis has also been associated with retinal vascular changes in hypertensive patients [17,27,28]. Specifically, one study found that patients with hypertension for more than 10 years have lower a peripapillary VD and perfusion density in the SCP. The authors did not find significant differences between the group with hypertension for less than 10 years and the control group [28]. Lim et al. [27] divided hypertensive patients by diagnosis for less than 5 years or greater than 5 years and found that patients with more than 5 years of hypertension had a thinner ganglion cell inner plexiform layer and peripapillary retinal nerve fiber layer compared to those of controls [27].

These preliminary findings suggest that the detection of capillary densities in the macula and peripapillary regions may serve as early pathologic markers of hypertensive damage and may ultimately improve abilities to monitor disease progression and better guide therapeutic treatment. For example, a study utilizing foveal OCTA in hypertensive patients found that foveal VD was significantly correlated with the Keith–Wagener–Barker grade of hypertensive retinopathy [29]. Of importance is that one study has found a positive moderate to strong correlation between retinal capillary density and cardiac remodeling markers in hypertensive patients [30]. It is important to note that, while consistent in the theme of outcomes, the current available data are limited and there is a lack of controlled studies accounting for treatments and comorbidities. The variation between studies on hypertension and OCTA biomarkers is likely due to differing OCTA machines and algorithms, and variations in patient populations, sample sizes, and outcome parameters (Table 1).

### 3.2. Atherosclerotic Disease

Atherosclerotic disease, a form of arteriosclerosis, is characterized by the thickening and hardening of artery walls due to the accumulation of fatty deposits, cholesterol, and other substances. This process leads to two major clinical categories: ischemic stroke and coronary artery disease (CAD). Ischemic stroke occurs when atherosclerotic plaques block blood vessels in the brain, leading to impaired blood flow and oxygen deprivation to brain tissues. This can result in brain damage and a loss of neurological functions. On the other hand, CAD involves the narrowing or blockage of the coronary arteries, which supply blood to the heart muscle. This can lead to angina (chest pain), myocardial infarction (heart attack), and other serious heart conditions. Both conditions are major contributors to morbidity and mortality worldwide and share common risk factors such as hypertension, high cholesterol, diabetes, obesity, and smoking.

The retina and choroid are affected by both CAD and ischemic stroke. Retinal and choroidal vascular biomarkers may act as powerful surrogates for measuring coronary circulation. Original studies [31,32] used fundus photography and retinal imaging to show a positive fair correlation between retinal arterial atherosclerosis and the severity of CAD [31].

Recent studies using OCTA have further characterized CAD’s effects on ophthalmic vasculature by identifying capillary dropout in the macula. Specifically, Wang et al. [33] found in a prospective, cross-sectional observational study that the mean VD of several layers, including both the SRL and DRL, was significantly lower in patients with CAD. Specifically, decreased VD and blood flow were associated with coronary artery and branch stenosis [33]. Another study examined 45 patients undergoing elective coronary angiography and OCTA. Patients with coronary heart disease confirmed using elective coronary angiography had a significantly lower VD in the SCP, DCP, and choriocapillaris [34]. Additionally, an observational study by Matuleviciute et al. [35] demonstrated a significantly decreased risk of three-vessel CAD with increased VD [35]. More recently, a study in patients with ST elevation myocardial infarctions found that decreased VD in the SCP, DCP, and choriocapillaris was positively correlated to left ventricular ejection fraction (LVEF), and SCP and DCP central fovea (1 mm) VD were negatively correlated to the number of affected coronary vessels in the studied patients [36]. 

While most studies (Table 2) demonstrated changes in both the SRL and DRL in patients with CAD, some studies demonstrated changes in the SRL alone. In 2018, Arounould et al. [37] studied changes only in the SRL. The study included 237 patients hospitalized for acute coronary syndrome and found that retinal VD in the SCP had a negative moderate correlation with higher American Heart Association risk and Global Registry of Acute Coronary scores, and lower left ventricular ejection fraction (LVEF) [37]. 

Some studies have sought to characterize retinal microvascular changes in patients with various levels of severity of coronary occlusion. Zhong et al. [38] observed retinal changes in patients with total coronary artery occlusion CAD (TCAO) on coronary angiography compared to those in patients with non-total coronary artery occlusion CAD (NTCAO) (>50% stenosis in at least one major artery). They found that VD of the SCP was significantly lower in TCAO patients versus those in NTCAO patients [38]. A different study in 2023 found that patients with obstructive coronary artery disease (OCAD) (>50% stenosis in at least one major coronary artery) and non-obstructive coronary artery disease (NOCAD) (20–50% stenosis in all major coronary arteries) had significantly reduced VD in all regions of the superficial vessel plexus and in all regions except for the fovea in the deep vessel plexus compared to the control. Additionally, a more significant decrease in VD was found in OCAD patients versus that in NOCAD patients [39]. 

The preponderance of study results suggests that significant changes in the retinal and choroidal microstructure and capillary loss occur in patients with CAD; thus, there is potential to use retinal vascular biomarkers for CAD early diagnosis, follow-up, and management. One study, however, by Arnould et al. [40] with 30 participants found no significant association between retinal VD and hemodynamic variables in the acute phase of myocardial infarction [40]. The scarce literature necessitates further investigation of the relationships between VD and CAD to elicit the specific prognostic and diagnostic abilities of each biomarker to disease outcomes.

The retinal and cerebral vasculatures share many similarities from embryology to functionality, thus allowing the non-invasive assessment of retinal vasculature to provide an estimation of changes in the cerebral vasculature. Previous studies have shown that changes in retinal blood vessels can be a window for evaluating cerebral vasculopathy [41,42]. With the advent of digital image processing, image analysis can be used to quantitatively measure retinal vasculature parameters such as central retinal artery equivalent diameter (CRAE), central retinal vein equivalent diameter (CRVE), and arteriovenous ratio (AVR), which is the ratio of the arteriole diameter to the venule diameter. These measurements have been used in many population-based studies to determine the relationship between retinal vessel characteristics and ischemic stroke [43,44,45].

Studies have shown that retinal vascular caliber has been independently correlated with stroke and can be viewed as an important marker for ischemic stroke detection [46,47]. Retinal caliber in patients with previous ischemic stroke was compared to that in control patients, and CRAE and subfoveal choroidal thickness in ischemic stroke patients were found to be significantly reduced compared to those in the control [43]. Highlighting the clinical potential of these findings, Zhao et al. [48] in 2021 developed a risk assessment model for ischemic stroke using measured retinal caliber and the Fazekas classification of white matter lesions along with traditional stroke risk factors in patients at risk for ischemic stroke. The authors found that the assessment model that included CRAE, the Fazekas classification of white matter lesions, and traditional risk factors was better than the single-index models they compared it to [48]. 

Studies investigated retinal structural and microvascular changes in ischemic strokes in patients with large artery atherosclerosis or small vessel occlusion (see Table 3). The superior peripapillary retinal nerve fiber layer thickness in large artery atherosclerosis patients was significantly thinner than that in small vessel occlusion patients [49]. A study by Duan et al. [50] found that vascular dropout may be a potential biomarker of ischemic stroke and its subtypes including lacunar and non-lacunar. The study demonstrated specific damage patterns in retinal microvascular and macular morphology in different subtypes of ischemic stroke. They found that lower vascular orientation distribution in the SCP was associated with lacunar infarction compared to non-lacunar infarction. In the DCP, however, they found that a higher FAZ axis ratio and lower FAZ circularity were associated with ischemic stroke, thus suggesting that the deep microvasculature could be more sensitive to ischemic stroke [50]. One study assessed patients with recent small subcortical infarcts and found that VD was significantly reduced in the superficial retinal capillary plexus and DCP of the macula compared to that in controls. They suggested that the DCP may be more susceptible to hypoperfusion because it is at the border of the inner plexiform layer and outer plexiform layer and therefore has a lower level of oxygen [51]. In contrast, a study in 2022 found that patients with thalamic infarcts had a significantly lower density in the SCP and intermediate capillary plexus compared to those in controls. No significant difference was found in the DCP between the thalamic infarct and control patients [52].

Multiple studies have found significant changes in both the SRL and DRL in patients with stroke. A case–control study with 15 ischemic stroke patients found that VD was significantly reduced at the level of all vascular plexuses in stroke patients [53]. Additionally, a study by Liang et al. [54] in 2022 found that the VD in the SCP and DCP was significantly reduced in ischemic stroke patients compared to those in the control [54]. Lu et al. [55] compared retinal microvasculature between ischemic stroke patients with large artery atherosclerosis (LAA) and small artery disease (SAD). They showed that LAA patients had significant capillary dropout in the deep vasculature compared with those with SAD; anterior LAA patients had significant capillary loss in the superficial retina compared with posterior LAA patients [55]. 

While the current literature regarding changes to the macular and peripapillary microvascular and ischemic stroke are variable and without consensus, the majority of OCTA studies suggest potential for its use to detect early retinal microvasculature changes in specific subtypes of ischemic stroke. However, only longitudinal studies inclusive of patients at high risk for various subtypes of ischemic stroke can provide clarity on the predictability of retinal VD outcomes for improved risk modeling and ultimately enhanced stroke patient care.

### 3.3. Congestive Heart Failure

Congestive heart failure (CHF) is a serious condition in which the heart fails to adequately perfuse the body. Although the pathogenesis of CHF is multifactorial, many studies have implicated a significant role in microvascular dysfunction [56,57,58]. Specifically, low cardiac output with CHF has been associated with compensatory mechanisms of peripheral vasoconstriction to maintain adequate BP and perfusion to essential tissues such as the heart and brain [59,60]. Some studies have found a decrease in cerebral blood flow in patients with CHF and have hypothesized a reduction in retinal blood flow as well [61,62]. 

Using Doppler ultrasound, Meira-Freitas et al. [63] evaluated the hemodynamics of the ophthalmic artery in patients with heart failure (HF) and proposed that HF could be a risk factor for low ocular perfusion [63]. In one of the first cross-sectional clinical studies to evaluate CHF and ocular blood flow via OCT, Altinkaynak et al. [64] demonstrated that the choroidal thickness in the macular area decreased in patients with CHF. Additionally, the authors found that the mean subfoveal choroidal thickness value was significantly lower in patients with CHF when compared with that in demographically matched healthy controls [64]. Using OCTA, Topaloglu et al. [65] found a significant lower VD in HF patients compared to that in controls (see Table 4) [65]. Although unable to show a statistically significant difference in flow densities between the study and control group, Alnawaiseh et al. [66] demonstrated a correlation between reduced macular flow density (whole en face) and LVEF, and New York Heart Association HF class, and showed a reduction in the ocular perfusion of chronic systolic HF when compared with that of healthy controls [66]. One study observed retinal VD using OCTA in children with HF due to dilated cardiomyopathy compared to that in controls and found that VD in the SCP was significantly lower in the HF group. No significant difference was found in the DCP or FAZ [67]. 

While not highly prevalent, some HF clinical studies are using retinal microvascular measurements as outcomes in patients [68]. The current findings indicate that OCTA and quantitative analyses of VD indeed may be useful as novel and non-invasive techniques to monitor microcirculation and central perfusion in patients with CHF. Long-term data are missing, however, and the prediction of OCTA biomarkers for CHF progression requires confirmation in significantly larger and well-controlled studies that specifically account for the impact of BP within this patient population.

## 4. Limitations

The limitations of this analysis include the diverse patient populations, variability in the inclusion of one or both eyes, limited OCTA data linked to cardiovascular disease, especially outside of the macular region, and lack of standardization in OCTA methodologies and data presentation. In addition, this analysis includes reports of average values for the whole region of interest assessed in the different studies (macula/peripapillary region) as means/ averages, and also regional differences (i.e., hemispheric/sectorial). Overall, well-controlled longitudinal studies with larger sample sizes are required to reach specificity in the location, significance, and pattern of retinal microvascular capillary dropout using OCTA for each one of these systemic diseases. Due to the complex nature of cardiovascular disease and BP, risk models should address and account for differences in BP and potentially hypertensive medication use among patient populations. 

## 5. Future Directions

As cardiovascular disease is a leading and rising cause of death globally, the improved non-invasive and highly accessible imaging of microvascular health will prevent a significant number of cardiovascular-related events, costs, and deaths. Well-controlled studies that account for the significant differences in blood and perfusion pressure across study populations and among cardiovascular disease groups are needed. In addition, long-term studies are needed to clarify acute versus chronic microvascular changes to reveal the strength and prognostic abilities over time. Artificial intelligence applications, including machine and transfer learning approaches to imagery, may reduce noise and increase the specificity and translatability of OCTA vascular biomarkers toward improved risk modeling and the enhanced management of systemic vascular disease.

## 6. Conclusions

The high prevalence and societal impact of systemic cardiovascular diseases necessitate improved diagnostic and predictive risk modeling approaches, with a focus on fast non-invasive acquisition and high accessibility. The eye is unique because it allows for the non-invasive and direct visualization and quantification of the earliest changes to human microvasculature. Advancements in OCTA imaging provide estimates of blood VD in specific regions of the retina and provide surrogate assessments of capillary loss and microvascular dropout. Although highly variable in the current literature, a preponderance of current studies has found lower retinal VD, suggesting that a reduction in microvascular capillaries may be associated with HTN, CAD, ischemic stroke, and CHF. 

The vast majority of studies have specifically identified microvascular changes occurring within the macular region, while significantly less is known about peripapillary and peripheral tissues. In addition, acute versus chronic relationships between retinal and systemic microvascular changes are almost entirely unexplored, and longitudinal data on disease progression are scarce. Well-controlled longitudinal studies are therefore needed to confirm these preliminary data, identify areas of earliest detectability, differentiate acute versus chronic changes to the retinal microvasculature, and clarify the exact prognostic abilities for each biomarker and its relationship to systemic cardiovascular disease. Additionally, as seen in Table 1, Table 2, Table 3 and Table 4, the calculation of the percent changes in OCTA VD was not possible in all the studies cited due to the limited available raw data, thus highlighting the need for well-designed, larger prospective controlled studies over time.

## Figures and Tables

**Table 1:** Summary of studies investigating retinal optical coherence tomography angiography (OCTA) parameters in subjects with systemic hypertension. Percent change of vascular density (VD) was estimated by subtracting the reported mean VD of the experimental group by that of the control group and dividing it by the VD of the control group. DCP: deep capillary plexus; DRL: deep retinal layer; DVP: deep vascular plexus; HTN: hypertension; SCP: superficial capillary plexus; SRL: superficial retinal layer; SVP: superficial vascular plexus.

	OCTA Device	Control Subjects/ Healthy	Patients Subject with Systemic HTN	Anatomic Region of the Retina with Capillary Dropout	OCTA Derived Parameter	Region of Interest/ Scan Type	Estimated Percentage of Dropout
Zeng et al. [18]	Zeiss CIRRUSTM HD-OCT Model 5000; Carl Zeiss Meditec Inc., Dublin, CA, USA	17	24	SCP	VD (%)	Macula (6 × 6 mm centered on fovea)	−2.86%
Terhedyen et al. [26]	Zeiss PLEX Elite 9000; Carl Zeiss Meditec, Dublin, CA, USA	31	28	DRL	VD (%)	Macula (cube of 3 × 3 mm centered on macula)	−16.50%
Sun et al. [24]	AngioVue: Optovue, Inc., Fremont, CA, USA	46	94	SVP, DVP	Macular Flow	Macula (6 × 6 mm centered on the fovea)	−23.33% (SVP) −9.60% (DVP)
Pascual-Prieto et al. [25]	RS-3000 AOCT Nidek Co., Gamagori, Japan	26	23	SCP, DCP	Macula perfusion density (%)	Macula (4.5 × 4.5 mm centered on fovea)	−11.46% (SCP) −4.88% (DCP)
Lee et al. [13]	Cirrus HD-OCT 5000 instrument (Carl Zeiss Meditec, Dublin, CA, USA) with AngioPlex software (ver.10.0; Carl Zeiss Meditec, Jena, Germany)	50	45	SRL	VD (%)	Macula (3 × 3 mm scan centered on the macula) Inner ring: (between a 1 and 3 mm diameter centered on the macula)	−5.85%
						Macula (3 × 3 mm scan centered on the macula) 3 mm full: 3 mm diameter centered on the macula	3.61%
Hua et al. [17]	OCTA using prototype AngioVue, software within the AngioVue device (RTVue XR Avanti with AngioVue, Optovue Inc., Fremont, CA, USA)	40	22	SVP	VD (%)	Macula (3.0 × 3.0-mm) Parafovea: (3 mm diameter circle centered on the macula)	−7.33%
Chua et al. [20]	AngioVue (Optovue, Inc., Fremont, CA, USA).	59 (well controlled BP)	18	DVP	Retinal Capillary Density (%)	Macula (6.0 × 6.0 mm^2^ field of view centered on the fovea)	−21.61%
Donati et al. [21]	AngioVue System on the RTVue XR Avanti device (Optovue, Inc., Fremont, CA, USA)	30	30	DVP	VD (%)	Macula (3 × 3 mm^2^ area) Parafoveal area (whole image minus foveal area).	−2.15%
						Whole macula (3 × 3 mm^2^ square area)	−2.33%
Lim et al. [27]	Zeiss Cirrus 5000 system (Carl Zeiss Meditec, Dublin, CA, USA)	117	52	SRL	VD (mm^−1^)	Macula (3 × 3 mm scan): 3 mm total area	−3.98%
Peng et al. [19]	RTVue-XR Avanti OCT System; Optovue, Inc., Fremont, CA, USA	30	113	SVP DVP	VD (%)	Macula (mean 6 × 6-mm^2^ )	−1.76 (SRL) −4.43% (DRL)
						Parafoveal area (a concentric circle 1 mm to the fovea)	−2.41% (SRL) −4.36% (DRL)
Sargues et al. [22]	Swept Source OCTA: SS-OCT Triton (Topcon, Tokyo, Japan) with automated OCTARA (OCT Angiography Ratio Analysis) algorithm provided by the device	71	93	DCP	VD (%)	Macula (4.5 × 4.5 mm) Parafovea (between external ring of 2.5 mm of diameter and a central area of 1 mm of diameter).	−1.54%
Xu et al. [23]	AngioVue system on the Avanti Spectral Domain OCT device (Optovue RTVue XR Avanti; Optovue Inc., Fremont, CA, USA).	79	137	DRL	VD (%)	Macula (6 × 6 mm) centered at the fovea	−9.69%

**Table 2:** Summary of studies investigating retinal optical coherence tomography angiography (OCTA) parameters in subjects with coronary artery disease. Percent change of vascular density (VD) was estimated by subtracting the reported mean VD of the experimental group by that of the control group and dividing it by the VD of the control group. CAD: coronary artery disease; CTO: coronary total occlusion; DCP: deep capillary plexus; DVP: deep vascular plexus; MI: myocardial infarction; OCAD: obstructive coronary artery disease; SCP: superficial capillary plexus; SRL: superficial retinal layer; SVP: superficial vascular plexus.

	OCTA Device	Control Subject/Healthy	Patients Subject with CAD	Anatomic Region of the Retina with Capillary Dropout	OCTA Derived Parameter	Region of Interest/ Scan Type	Estimated Percentage of Dropout
Matuleviciute et al. [35]	OCT DRI OCT Triton (Topcon, Tokyo, Japan)	82	26 (3 vessel disease, 76 MI)	SCP, DCP	VD (%)	Macula (3 × 3 and 6 × 6 mm images of central macular area)	Data presented as OR
Aschauer et al. [34]	PLEX Elite 9000 (Carl Zeiss Meditec)	18	27	SCP, DCP	VD (%)	Macula (6 × 6 fovea centered)	Raw data not presented
Arnould et al. [37]	CIRRUS HD-OCT, Model 5000; Carl Zeiss Meditec AG with Angiography software (Angioplex, version 10; Carl Zeiss Meditec AG)	44	237 CAD	SCP	VD (%)	Macula (3 × 3 mm)	Raw data not presented
Zhong et al. [38]	RTVue-XR Avanti; Optovue, Fremont, CA, USA	116	102 CTO with CAD	SRL	VD (%)	Macula mean (6 × 6 mm^2^)	−4.17%
						Parafoveal (area between 1–3 mm concentric ring centered on the fovea)	−5.67%
Ren et al. [39]	AngioVue (RTVue-XR Avanti; Optovue, Fremont, CA, USA	58	62 (OCAD)	SVP, DVP	VD (%)	Parafoveal (region between the 1–3 mm concentric ring center of the fovea)	−13.14% (SVP) −9.55% (DVP)
						Macula whole (6 × 6 mm^2^)	−11.93% (SVP) −13.85% (DVP)
Sideri et al. [36]	DRI OCT Triton Swept Source Optical Coherence Tomograh (Topcon, Tokyo, Japan).	77	88	SCP, DCP	VD (%)	Macula (3 × 3 mm centered on the fovea) perifoveal (are between the 1–3 mm)	Complete raw data not provided for control group

**Table 3:** Summary of studies investigating retinal optical coherence tomography angiography (OCTA) parameters in subjects with ischemic stroke. The percent change of vascular density (VD) was estimated by subtracting the reported mean VD of the experimental group by that of the control group and dividing it by the VD of the control group. DCP: deep capillary plexus; DRL: deep retinal layer; DVP: deep vascular plexus; IPR: inner retina plexus; RPCP: radial peripapillary capillary plexus; SCP: superficial capillary plexus; SVP: superficial vascular plexus.

	OCTA Device	Control Subject/Healthy	Patients Subject with Ischemic Stroke	Anatomic Region of the Retina with Capillary Dropout	OCTA Derived Parameter	Region of Interest/ Scan Type	Estimated Percentage of Dropout
Duan et al. [50]	AngioVue, RTVue XR Avanti spectral domain OCT, Optovue, Fremont, CA, USA	43	52	DRL	Vascular Area Density	Macula (3 × 3 centered at the fovea)	Data presented as OR
Cao et al. [51]	Avanti RTVue-XR tool (Optovue, Fremont, CA, USA;)	46	40	SRCP, DRCP	VD (%)	Macula (information not provided; whole)	−4.83% (SRCP) −6.26% (DRCP)
Zhang et al. [49]	RTVue XR with AngioVue (software version 2017.1.0.155; Optovue, Inc., Fremont, CA, USA).	65	69	DCP	VD (%)	Macula (3 × 3 mm^2^ around the fovea)	−3.98% (LAA) −2.73% (SAA)
Ye et al. [52]	VG 200; SVision Imaging Limited Luoyang, China)	31	35	SVP	VD (%)	Macula (3 × 3 mm annulus around the fovea)	Raw data not provided
Molero-Senosiain et al. [53]	RS-3000, Nidek Co.	50	15	RPCP + SCP + IPR, DCP	VD (%)	Macula (4.5 × 4.5 mm)	−24.93% (RPCP + SCP + IPR) −33.82% (DVP)
Liang et al. [54]	RTVue-XR Avanti; Optovue	109	159	SCP, DCP	VD (%)	Whole macula (6 × 6 mm^2^)	−3.82% (SCP) −6.03 (DCP)
						Parafovea (1- to 3 mm annulus foveal region)	−5.13 (SCP) −5.04 (DCP)

**Table 4:** Summary of studies investigating retinal optical coherence tomography angiography (OCTA) parameters in subjects with congestive heart failure (CHF). The percent change of vascular density (VD) was estimated by subtracting the reported mean VD of the experimental group by that of the control group and dividing it by the VD of the control group. DCP: deep capillary plexus; NYHA: New York Heart Association; SCP: superficial capillary plexus.

	OCTA Device	Control Subjects/ Healthy	Patients Subject with CHF	Anatomic Region of the Retina with Capillary Dropout	OCTA Derived Parameter	Region of Interest/ Scan Type	Estimated Percentage of Dropout
Rakusiewicz et al. [67]	RTVue-XR (Angiovue; Optovue Inc., Fremont, CA, USA).	30	30	SCP	VD (%)	Whole macula (3 × 3 mm area centered at fovea)	−7.28%
						Parafoveal (ring with an internal diameter of 1 mm and external of 3 mm)	−6.23%
Topaloglu et al. [65]	DRI OCT (Triton, Topcon, Tokyo, Japan)	36	18 (NYHA III)	DCP	Capillary Plexus Perfusion	Macula (3 × 3 mm^2^)	−15.27%

## Data Availability

No new data were created or analyzed in this study. Data sharing is not applicable to this article.

## Abbreviations

AVR	Arteriovenous Ratio
CAD	Coronary Artery Disease
CHD	Coronary Heart Disease
CHF	Congestive Heart Failure
CRAE	Central Retinal Artery Equivalent
CRVE	Central Retinal Vein Equivalent
CTO	Coronary Total Occlusion
CVD	Cardiovascular Disease
DCP	Deep Capillary/Vascular Plexus
DRL	Deep Retinal Layer
DVP	Deep Vascular Plexus
FAZ	Foveal Avascular Zone
HF	Heart Failure
HTN	Hypertension
IS	Ischemic Stroke
LAA	Large Artery Atherosclerosis
LVEF	Left Ventricular Ejection Fraction
MI	Myocardial Infarction
NYHA	New York Heart Association
OCTA	Optical Coherence Tomography Angiography
SAD	Small Artery Disease
SCP	Superficial Capillary Plexus
SRL	Superficial Retinal Layer
SVP	Superficial Vascular Plexus
VD	Vascular Density
